# Limited effects of tannin supplementation on the dairy cattle fecal microbiome with modulation of metabolites

**DOI:** 10.3389/fmicb.2025.1570127

**Published:** 2025-06-10

**Authors:** Matthew L. Klein, Christian B. Erikson, Conor J. McCabe, Laibin Huang, Jorge L. Mazza Rodrigues, Frank M. Mitloehner

**Affiliations:** ^1^Department of Animal Science, University of California, Davis, Davis, CA, United States; ^2^Department of Land, Air, and Water Resources, University of California, Davis, Davis, CA, United States; ^3^Department of Biology, Saint Louis University, St. Louis, MO, United States; ^4^Environmental Genomics and Systems Biology Division, Lawrence Berkeley National Laboratory, Berkeley, CA, United States

**Keywords:** quebracho, tannins, microbiome, cattle, metabolites, indole, feed additive

## Abstract

Tannins are plant secondary metabolites that bind organic carbon (C) and nitrogen (N), potentially altering substrate bioavailability for enteric fermentation in ruminants. This interaction may reduce greenhouse gas (GHG) emissions and influence nitrogen partitioning. Given tannins' resistance to ruminal degradation and persistence through the gastrointestinal tract, this study investigated the effects of a tannin-based feed additive on fecal microbial diversity, fecal chemical composition, and GHG emissions. Twenty-four early- to mid-lactation dairy cows were randomized to receive either a tannin-based feed additive (TRT; containing condensed and hydrolyzable tannins from *Schinopsis quebracho-colorado* [Schltdl.]) or a control diet (CON) for 64 days. Cows were blocked by parity, dry matter intake, milk yield, body weight, and days in milk. Fecal samples were collected on days 0, 16, 32, and 64 and analyzed using 16S rRNA gene amplicon sequencing. Fecal C, N, and indole-3-lactate were measured, and GHG emissions (N_2_O, CH_4_, CO_2_) were assessed via 14-day laboratory incubation. A total of 1,538 amplicon sequence variants were identified, with Firmicutes as the dominant phylum. Fecal phylogenetic diversity showed a significant treatment × day interaction (*p* < 0.01), with TRT cows exhibiting reduced microbial diversity from day 16 to 64. Fecal C and N concentrations were significantly lower (*p* < 0.01) in TRT cows on day 16, while indole-3-lactate levels were higher on day 64 (*p* = 0.02). GHG emissions did not differ significantly between treatments. The tannin-based feed additive influenced fecal microbial community structure and select chemical parameters but did not significantly affect GHG emissions from feces. These findings suggest that dietary tannins may modulate gut microbial ecology with minimal impact on downstream manure-related emissions.

## 1 Introduction

The ruminant digestive system symbiotically allows both ruminant hosts and gut microbes to exploit the enteric fermentation of plant biomass for nutrition and energy (Ley et al., 2008; Mizrahi et al., 2021). However, enteric fermentation leads to the production of methane (CH_4_), a greenhouse gas (GHG) with a global warming potential (GWP) 28 times that of CO_2_ (EPA, 2024). Dairy cattle are a substantial source of CH_4_, accounting for 8.2 and 5.6% of agricultural GHG emissions in the United States via enteric fermentation and manure management, respectively (EPA, 2024). Therefore, strategies that improve efficiency are of utmost importance in allowing ruminant production to meet environmental standards. Feed additives are one such strategy that is gaining widespread recognition as a tool to reduce CH_4_ emissions (FAO, 2023). The complex microbial environment of the rumen can respond in numerous ways to the introduction of a new feed ingredient; as such, targeting nitrogen (N) metabolism can be a co-benefit of feed additives alongside CH_4_ abatement.

Ruminants retain ~20% (range: 15%−40%) of ingested N as meat or milk, meaning the ruminant excretes the majority of dietary N in manure (Kohn et al., 2005; Huhtanen and Hristov, 2009). Manure N can then undergo denitrification, which yields the GHG nitrous oxide (N_2_O) (Robertson and Groffman, 2015), which has a GWP 273 times that of CO_2_ (IPCC, 2021). Dairy cattle in the United States account for 1% of agricultural emissions in the form of N_2_O from manure management, and ~2.5% of US agricultural emissions in the form of N_2_O from agricultural soil management, i.e., manure fertilizer application (EPA, 2024).

Due to their unique chemistry, condensed tannins (CT), a class of plant secondary metabolite, can inhibit rumen microbes, reduce enteric methanogenesis, and impact diet digestibility, and are therefore considered a dietary means of reducing enteric and manure GHG emissions (Aboagye and Beauchemin, 2019; Ingold et al., 2021; Zhao et al., 2023). Condensed tannins bind to protein in the rumen, forming indigestible complexes that are hydrolyzed by the acidic pH of the abomasum, where the protein is then released in the lower gastrointestinal tract (GIT), which can lead to increased N excretion in feces (Jones and Mangan, 1977; Hagerman et al., 1992; Patra and Saxena, 2011; Aboagye and Beauchemin, 2019). Tannins also bind to various carbohydrates, including hemicelluloses, cellulose, starch, and pectin (Besharati et al., 2022), and are known to complex with metals, including copper and zinc (Kraus et al., 2003; Karamać, 2009).

By modulating microbial access to dietary protein, CT can also affect the production of microbial amino acid-derived metabolites such as skatole and *p*-cresol, putrescine, and various indoles (Ehrlich et al., 2020; Gasaly et al., 2021; Gasaly and Gotteland, 2022). By modulating the production of these metabolites, CT administration could lead to benefits beyond GHG reductions, since, for example, various indoles have been shown to have anti-inflammatory effects on the host animal (Gasaly et al., 2021; Gasaly and Gotteland, 2022). However, at higher doses of >2%−3% dry matter (DM), dietary tannins can exhibit distinct anti-nutritive properties, by decreasing both diet digestibility and nutrient density (Koenig and Beauchemin, 2018; Aboagye and Beauchemin, 2019). In a review of 58 dairy farms, dietary total crude protein (N × 6.25) digestibility was reduced by 5% for each 0.9% DM tannins in the diets (Herremans et al., 2020). Therefore, precise administration of low to moderate concentrations is required to enable improved digestive efficiency and microbial community function while minimizing the anti-nutrient downsides (Frutos et al., 2004). Rumen microbes also exhibit limited ability to degrade tannins (Makkar et al., 1995; McSweeney et al., 2001), which means that unmetabolized tannins pass through the digestive tract to continue having bioactive effects in feces (van Cleef et al., 2022).

Given the well-documented C and N binding ability of tannins, we hypothesized that a feed additive treatment, a mixture of condensed tannins extracted from quebracho trees [TRT; *Schinopsis quebracho-colorado* (Schltdl.)], when included in a dairy cow diet, would affect the fecal microbial community, modulate fecal chemical properties and concentrations of microbial amino acid-derived metabolites, and decrease emissions of N_2_O, CH_4_, and CO_2_ from feces. The objectives of this study were to: (1) determine differences in fecal microbial communities, through 16S rRNA gene amplicon sequencing of TRT (a top-dressed CT-based feed additive added to the standard diet at 0.15% DM) and control (CON; un-supplemented dairy diet) fecal samples, (2) determine the extent to which TRT-modified fecal chemical properties, including microbial amino acid-derived metabolites, and (3) determine greenhouse gas emission fluxes of feces from TRT vs. CON dairy fecal samples.

## 2 Materials and methods

### 2.1 Sample collection and study herd

Animals used in this study were housed at the University of California, Davis, Dairy Teaching and Research Facility under research protocol number 22348, approved by the Institutional Animal Care and Use Committee (IACUC). Treatment animals were administered a feed additive containing a blend of condensed and hydrolyzable tannins extracted from quebracho (70% w/w tannic acid).

The overall experiment was designed as a completely randomized block design with repeated measures wherein 24 mid-lactation Holstein cows (12 controls; CON, 12 treatments; TRT; *n* = 12) were randomized to TRT or CON. Two cows were paired in each block, with each block consisting of one TRT-fed and one CON-fed cow. Cows were also blocked by parity, dry matter intake, milk yield, body weight, and days in milk, and housed in a pen equipped with a Calan Broadbent Feeding System (American Calan, Northwood, New Hampshire) to allow for individual feeding. Cows on TRT were fed the feed additive as a top dress at a rate of 0.15% dry matter (DM) for a 64-day study period, and enteric greenhouse gases were measured with the head chamber system described by Place et al. (2011). Furthermore, to account for two cows being sampled for gas emission per day in the two head chambers (Place et al., 2011), cow blocks were stagger-started onto their respective treatments.

Animals were contained in the head chambers for 2 h at a time, followed by 4 h in the group pen, for a total of 8 h per day of sampling time on each animal in the head chambers. During their stay in the head chambers, total feces were collected directly into plastic bags for each cow, which were homogenized with a handheld mixer, and 50 ml subsamples were collected in triplicate and stored at −20°C until further processing. Fecal samples for each cow were collected on days 0, 16, 32, and 64 for 96 samples. The experimental total mixed ration (TMR) was formulated to meet the nutritional requirements of mid-lactation dairy cattle following the National Research Council guidelines (NRC, 2021). The DM and crude protein contents of the diet were 87.7 ± 0.86% DM and 17.2 ± 0.12% DM, respectively (Cumberland Valley Analytical Services; Waynesboro, PA, USA). The daily offered amount of the TMR was adjusted to allow 10% orts on an as-fed basis, according to intake observed on the previous day.

### 2.2 DNA extraction and 16S rRNA gene sequencing

The sample DNA was extracted from composite fecal samples collected during each cow's stay in the head chambers. Total DNA was extracted from 0.25 mg of homogenized fecal samples using DNeasy^®^ PowerLyzer^®^ PowerSoil^®^ Kit (QIAGEN, Hilden, Germany). The DNA was quantified using the Qubit 3.0 device (Thermo Fisher Scientific, Waltham, MA, USA). Initial extracts contained 13.48 ± 0.67 ng/μl of DNA prior to purification and PCR. Bacterial and archaeal abundances were determined by amplifying the V4 region of the 16S rRNA gene using forward 515F and reverse barcoded 806R primers (Caporaso et al., 2011). Individual indices can be seen in Supplementary Table S1. Extracted DNA was stored at −20°C prior to PCR amplification, which was carried out using the Phusion Hot Start II High-Fidelity PCR Master Mix (Thermo Fisher Scientific, Waltham, MA, USA). A PCR assay was conducted in triplicate, and samples were combined and quantified by Qubit 3.0 with dsDNA high sensitivity reagents. Each sample was then pooled with equimolar concentrations (100 ng DNA/sample) into a single sterile tube and purified using the QIAquick PCR Purification Kit (QIAGEN, Hilden, Germany). The DNA was visualized on a 1.5% agarose gel for quality assurance, and nuclease-free water was also amplified with samples and sequenced as a control for kit contamination. The final library was sequenced at the DNA Technologies Core Facility of the Genome Center at the University of California, Davis, on the Illumina MiSeq platform using MiSeq Reagent Kit v2 in 250 bp paired-end mode.

### 2.3 Amplicon sequence processing

Sequence processing on raw FASTQ files received from the DNA Technologies Core Facility began with a generalized DADA2 workflow written in snakemake (retrieve from: https://github.com/cErikson/GeneLab_DADA2_snakemake_Pipeline), which was adapted from Callahan et al. (2016a) and Callahan et al. (2016b). Adapter trimming was performed with cutadapt, using custom adapter sequences, and the non-default settings of max-n: 0, qual_trim_5: 2, qual_trim_3: 25 trim_end_n: True. Filtering of reads was performed by the DADA2 filter function with the non-default parameters of maxee:2 and truncq:2. Interactive plots were generated and data quality inspected with MultiQC (Ewels et al., 2016). Based on all quality control steps, all sequences were trimmed to 252 bp. Taxonomic assignment was performed with the Silva NR 99 SSU version 138.1 database, with clustering at 99% identity (Quast et al., 2013). A phylogenetic tree was constructed with MAFFT using -auto and FastTree with default parameters (Katoh et al., 2002). The generated amplicon sequence variant (ASV) table, taxonomy table, phylogenetic tree, and sample metadata were imported into the R package Phyloseq (McMurdie and Holmes, 2013). In R, several final filtering steps were conducted, followed by all downstream analyses. Filtering removed all ASVs without a taxonomic assignment at the class level, any ASVs labeled as mitochondria, chloroplast, or eukaryote, and any classifications with “na.” The *tax_filter* function from the package MicroViz was used for a prevalence filter; “min_prevalence” was set at 0.03, and “prev_detection_threshold” was set at 3 (Barnett et al., 2021). With the package vegan, samples were rarified to 29,090 reads per sample, a depth of 90% that of the minimum sample (Oksanen et al., 2022). To check for outliers, principal coordinate analysis (PCoA) was performed with Bray's distances on log-transformed data, with ordination plots by treatment and by phylum. No outliers were identified in the amplicon sequence data or removed based on this assessment.

### 2.4 Microbial community analysis and statistics

All statistical analyses were conducted in R (v 4.3.1) on pre-processed data (R Core Team, 2023). Relative abundance of phyla was statistically analyzed with the Wilcoxon rank-sum test for each phylum, with a false discovery rate (FDR) corrected *p*-value of ≤ 0.05 being significant. Further differential abundance analysis at lower taxonomic ranks was carried out with the *differentialTest* function in the corncob package (Martin et al., 2024). Model settings of “formula = ~ treatment × day,” “phi.formula = ~ treatment × day,” “formula_null = ~ 1,” and “phi.formula_null = ~ day” were used to determine any interaction effects over the 64-day study. An FDR cutoff of ≤ 0.05 was considered significant. The model was implemented with default parameters, and as such, only accounted for the specified treatment × day interaction with no further block or covariates included in the model.

Alpha-diversity was analyzed with Faith's phylogenetic diversity (PD; Faith, 1992). The following model was implemented for both alpha and beta diversity statistical analyses:


(1) $$\begin{array}{l} Y_{ijkl}=\;u\;+\;t_{i}\;\times \;day_{j}+block_{k}+{\left(1|cow\right)}_{l}+bl+\varepsilon_{ijkl} \end{array}$$

where *Y* is the response variable, *t*_i_ is the fixed effect of the feed additive treatment where i is either TRT or CON, day_j_ is the fixed effect of sampling day, where *j* = 16, 32, or 64 days following treatment administration, block_k_ is the fixed effect of block where animals were grouped into *k* blocks (12 blocks of two cows in each block), (1|cow)_l_ is the random effect of cow, bl is the baseline day 0 measurement of the response variable included as a covariate, and *e*_ijkl_ is the residual error term. Additional alpha diversity metrics, including Observed Richness, Simpson's index, and Shannon's index, were calculated for days 16 to 64. The *alpha* function in the microbiome R package was used for these calculations (Lahti, 2023), while PD was calculated with the *pd* function in the Picante package (Kembel et al., 2010). Alpha diversity metrics were checked for normality via the *qqPlot* function, and homogeneity of variance was verified with a plot of residuals vs. fitted values. Alpha diversity metrics were then statistically analyzed via the *lmer* function (Bates et al., 2015). Pairwise comparisons of alpha diversity by treatment and day were conducted via *emmeans* (Lenth et al., 2023). To investigate beta diversity, i.e., the differences in the overall microbial community composition, weighted UniFrac distances were calculated using the Phyloseq function, *ordinate*. To visualize the overall microbial composition by treatment × day, PCoA plots were generated on the weighted UniFrac distances using the *plot_ordination* function found in Phyloseq. Significance of clustering via PERMANOVA was tested using the *adonis2* function with 1,000 permutations (Oksanen et al., 2022). Additionally, the longitudinal effects on weighted UniFrac distances, Bray–Curtis dissimilarity, Jaccard distance, and Jensen–Shannon divergence were further assessed with the MicrobiomeStat package and the function *generate_beta_change_per_time_test_long* (Yang et al., 2025), which allowed for implementing Equation 1 described above.

To further evaluate whether taxa at the family level correlated with fecal chemical properties (chemical analyses described below), Kendall's tau correlation coefficients and *q-*values were calculated for each family × chemical analyte with the *cor_test* function from the microViz package (Barnett et al., 2021). These correlations were performed on TRT and CON data for days 16, 32, and 64 and did not include any covariates (i.e., day 0, block). The coefficient *p-*values were adjusted by the Benjamini–Hochberg FDR, with significant associations being considered at *q* < 0.05. In the final plot, asterisks were used to indicate a *p*-value of < 0.05 and filled circles to indicate *q* < 0.05. The Euclidean dendrogram distances were calculated with the *dist* function, and were clustered with the “Ward.D2” option in the *hclust* function, both found in the base R package, stats (R Core Team, 2023). Coefficients were then plotted by treatment, utilizing *ggplot* (Wickham, 2016) and the *scale_(x/y)_dendrogram* function from the ggh4x package (van den Brand, 2024).

### 2.5 Fecal chemical properties, metabolites, and greenhouse gas emissions

All fecal samples were also analyzed in triplicate for total C, organic N, C:N ratio, DM, pH, phosphorus, potassium, copper, iron, zinc, and soluble salts by Ward Laboratories, Inc., Kearney, NE. Minerals, including copper and zinc, were quantified with initial hydrochloric acid and nitric acid digestion followed by inductively coupled plasma optical emission spectroscopy [Campbell and Plank, 1991; Kovar, 2003; Peters et al., 2003; Association of Analytical Chemists (AOAC), 2019]. Fecal samples were also processed at the University of California, Davis West Coast Metabolomics Center for microbial amino acid-derived metabolites. Homogenized subsamples (0.02 g) were analyzed along with appropriate standards via gas chromatography time-of-flight mass spectrometry (Pegasus BT, LECO Corporation, St. Joseph, MI). Raw metabolite data can be seen in Supplementary Table S2. A specific subset of metabolites was of interest, given the nature of the animal system and feed additive, namely, *p*-cresol, skatole, indole-3-lactate, indole-3-acetate, putrescine, spermine, and spermidine were measured. Chemical properties and metabolites were statistically analyzed in R with the *lmer* function, and the model:


(2) $$\begin{array}{l} Y_{ijklm}=\;u\;+\;t_{i}\;\times \;day_{j}+block_{k}+{\left(1|cow\right)}_{l}+{\left(1|replicate\right)}_{m}\\ \;+\;bl\;+\;\varepsilon_{ijklm} \end{array}$$

where variables were the same as described for Equation 1 except (1|replicate)_m_ was included as a random effect of replicate. Pairwise comparisons were made with the *emmeans* function. Influential points were assessed against a Cook's distance >0.5. Several influential points/potential outliers were subsequently identified and assessed for their influence on organic N and indole-3-lactate statistical results.

Greenhouse gas emissions from feces were measured in a 14-day laboratory incubation. On days 64, 25 g DM of homogenized feces samples were collected in triplicate from each cow in both the TRT and CON groups. Each 25 g sample was placed into an individual 100 ml specimen cup housed within a glass 1-L Mason jar fitted with a 1.5 cm diameter sponge-ventilated lid to allow for air flow. This laboratory incubation design has been described previously by Zhu et al. (2013) and Ellison and Horwath (2021) and is widely applicable to organic materials, including soils, manures, and organic wastes. The incubation was organized as a completely randomized block design in triplicate with repeated measures. Humidity inside the jars was maintained by the addition of 2 ml of DI water to the bottom of the jar, and all jars were housed at 22°C. For GHG measurements, made on days 1, 2, 3, 5, 7, and 14 of the incubation, the 1-L Mason jars were sealed with lids fitted with butyl septa (Supelco, Sigma-Aldrich, St. Louis, MO) and 20 ml headspace gas samples were taken at 0-, 30-, and 60-min after sealing jars. The concentrations of N_2_O, CH_4_, and CO_2_ were measured by gas chromatography (Model GC 2014, Shimadzu Scientific Instruments, Kyoto, Japan).

Estimated daily GHG fluxes were determined by linear interpolation of the 0-, 30-, and 60-min measurements. Average daily GHGs were calculated with an assumption that the interpolated 1-h flux previously described was representative of an average daily flux (Ellison and Horwath, 2021). Differences in average daily fluxes (averaged over replicates within each day) were analyzed with the *lme* function and the model:


(3) $$\begin{array}{l} Y_{ijk}=\;u\;+\;t_{i}\;\times \;day_{j}+{\left(1|block/cow\right)}_{k}+\varepsilon_{ijk} \end{array}$$

where *Y* is the GHG response variable, *t*_i_ × day_j_ are treatment and day as described above, and (1|block/cow)_k_ is the random effects of block and cow, with a varying intercept among block and cow within block. The *emmeans* function was subsequently used for mean comparisons. All figures incorporated the use of the ggplot2 package (Wickham, 2016).

## 3 Results

### 3.1 Sequence data processing

The single run of MiSeq PE 250 returned 14.6 million paired-end reads that passed the Illumina control filter with an overall Q30 > 84.5%. Before filtering in R, sequences were all adapter-trimmed, and the library size following DADA2 processing ranged from 34,050 to 60,442 reads with a median library size of 47,524 reads. After filtering, the library size for the samples varied from 32,323 to 57,429 reads, with a median library size of 45,029 reads. The samples were rarified to 90% of the minimum filtered library size, which resulted in 29,090 reads per sample. Clustering in DADA2 yielded a total of 13,259 ASVs, of which 11,721 ASVs were removed through several filtering steps, i.e., incomplete assignment, low prevalence. The control samples contained a total of 1,139 reads, indicating only minor contamination, so the controls were not statistically analyzed further. The resulting final ASV table had 1,538 ASVs across 96 samples.

### 3.2 Tannin feed-additive impact on microbial diversity

**Figure 1:**
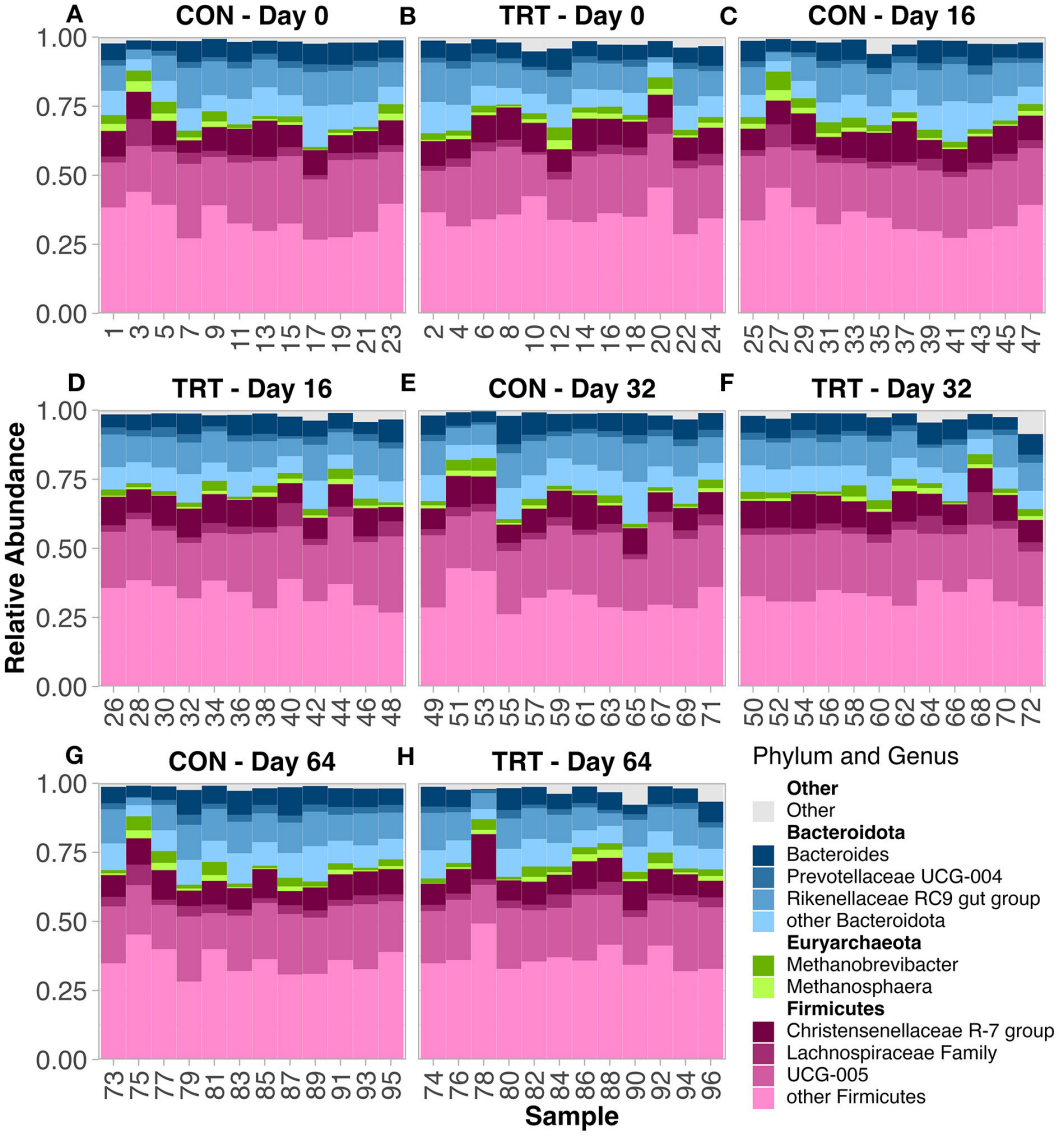
Relative abundance of the prokaryotic community in fecal samples from dairy cows fed a control (CON) diet vs. a tannin treatment (TRT) diet containing a 0.15% dry matter (DM) blend of quebracho tannins at 0-, 16-, 32-, and 64-days following TRT administration. **(A–H)** relative abundance by Cow ID. **(A)** TRT—Day 0, **(B)** CON—Day 0, **(C)** TRT—Day 16, **(D)** CON—Day 16, **(E)** TRT—Day 32, **(F)** CON—Day 32, **(G)** TRT—Day 64, **(H)** CON—Day 64.

**Figure 2:**
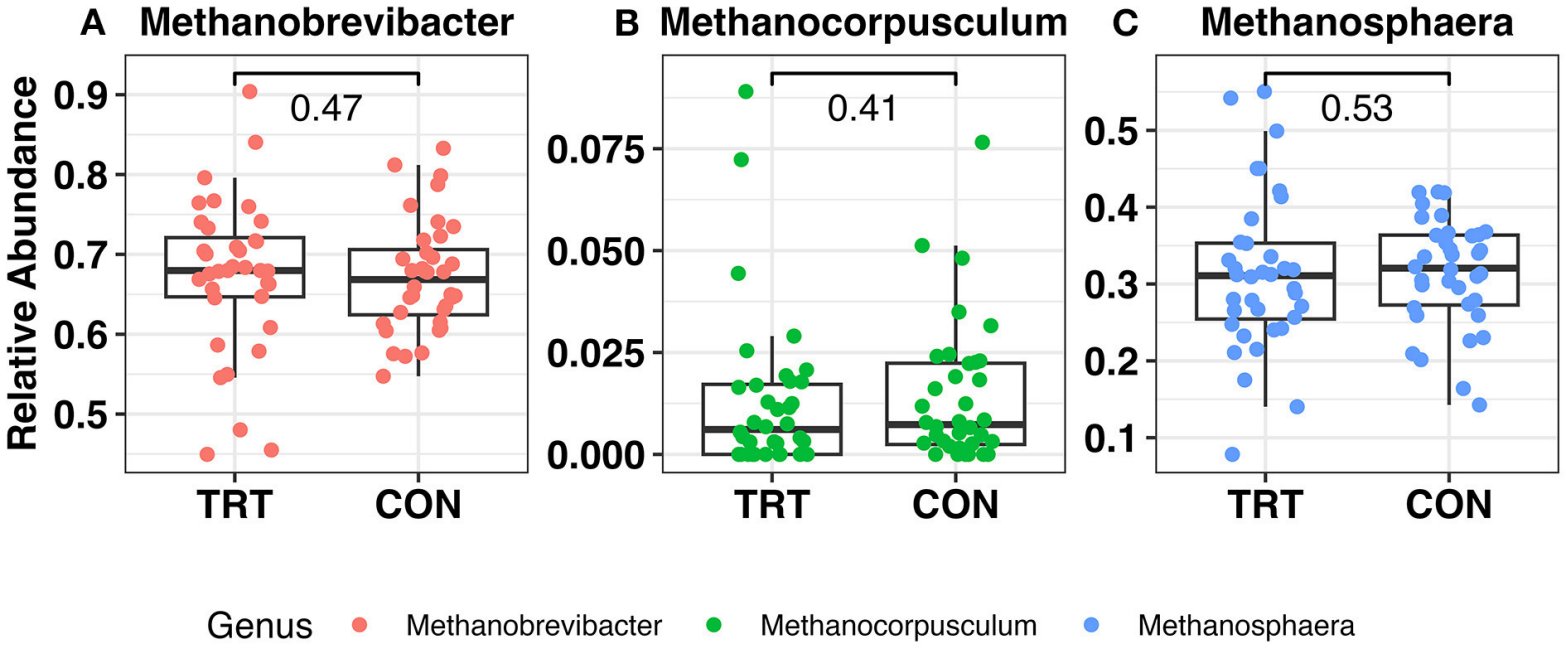
Relative abundance of the methanogen community in fecal samples from dairy cows fed a control (CON) diet vs. a tannin treatment (TRT) diet containing a 0.15% dry matter (DM) blend of quebracho tannins at 0, 16, 32, and 64 days following TRT administration. **(A)** *Methanobrevibacter*, **(B)** *Methanocorpusculum*, **(C)** *Methanosphaera*.

There were 13 phyla observed across all samples. The microbial community was predominantly classified as Firmicutes or Bacteroidota, which summed to ~92%−93% of the microbial community in both TRT and CON cow fecal samples, respectively (Figures 1A–H; Table 1). The phylum level composition of the TRT vs. CON cow fecal samples was similar across the duration of the study, and according to the FDR controlled Wilcoxon rank-sum test (Table 1), the only difference in relative abundance at the phylum level was found for Actinobacteriota which was significantly greater in TRT vs. CON cow fecal samples on day 0 (*p* = 0.04) and across days 16–64 (*p* = 0.02). The relative abundance of methanogen genera was also specifically analyzed, with *Methanobrevibacter, Methanocorpusculum*, and *Methanosphaera* being detected, however, with no differences in abundance (Figures 2A–C).

**Table 1:** Average relative abundance of the fecal microbial community across days 16 to 64, from dairy cows fed a control (CON) diet vs. a treatment (TRT) diet containing a 0.15% dry matter (DM) blend of quebracho tannins (*n* = 48).

**Phylum**	**Average relative abundance (%)^a^**		
	**TRT**	**CON**	***p*** **-Value^b^**
Firmicutes	67.76 ± 4.39	66.62 ± 5.22	0.62
Bacteroidota	25.59 ± 4.76	27.56 ± 6.40	0.94
Euryarchaeota	3.36 ± 1.51	3.70 ± 2.16	0.85
Actinobacteriota	1.84 ± 1.79	0.85 ± 0.64	0.02
Spirochaetota	0.72 ± 0.02	0.86 ± 0.53	0.85
Verrucomicrobiota	0.19 ± 0.14	0.20 ± 0.13	0.85
Proteobacteria	0.18 ± 0.18	0.14 ± 0.12	0.67
Halobacterota	0.03 ± 0.03	0.04 ± 0.04	0.94
Fibrobacterota	0.009 ± 0.02	0.01 ± 0.03	0.85
Planctomycetota	0.006 ± 0.01	0.003 ± 0.008	0.62
Chloroflexi	0.003 ± 0.007	0.003 ± 0.005	0.66
Desulfobacterota	0.001 ± 0.003	< 0.001 ± 0.003	0.66
Elusimicrobiota	< 0.001 ± 0.001	< 0.001 ± 0.002	0.85
Firmicutes/Bacteroidota ratio	2.65:1 ± 4.56	2.42:1 ± 5.81	0.76

a Average relative abundance ± standard deviation of classified phyla after filtering data.

b FDR corrected p-values derived from the Wilcoxon rank-sum test.

**Figure 3:**
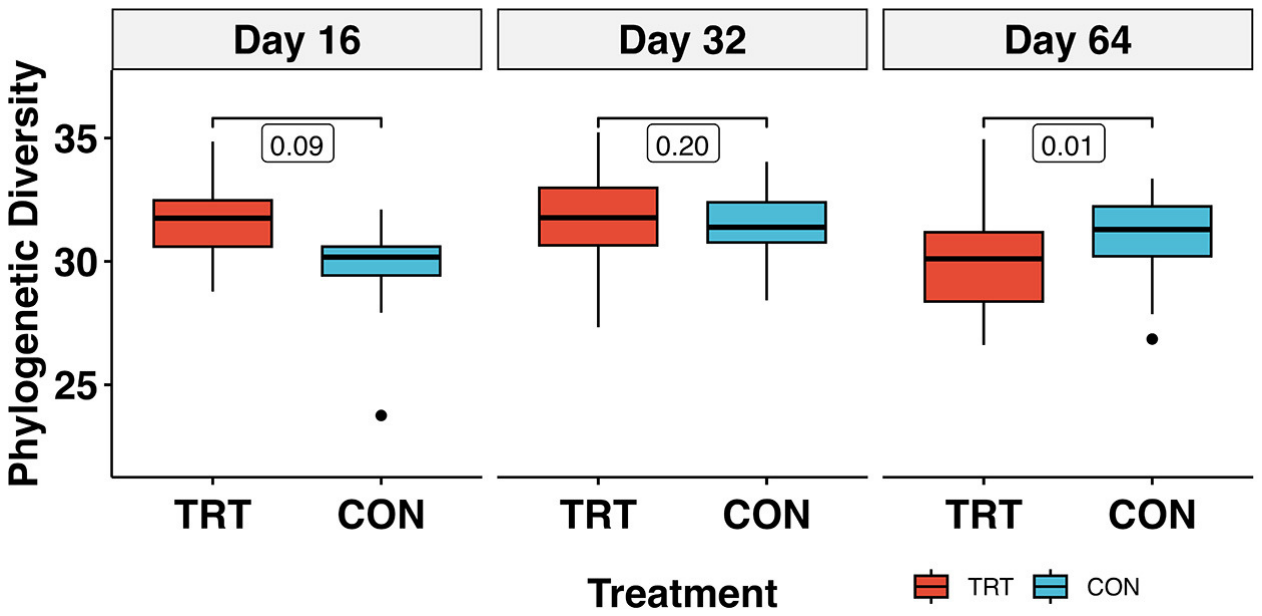
Faith's phylogenetic diversity of the prokaryotic community in fecal samples from dairy cows fed a control (CON) diet vs. a tannin treatment (TRT) diet containing a 0.15% dry matter (DM) blend of quebracho tannins at 0, 16, 32, and 64 days following TRT administration^1^. ^1^Bracketed values represent *p*-values from emmeans; model results are in Table 2.

The alpha diversity metric PD returned a treatment × day interaction (*p* < 0.01) when analyzed via *lmer*. Pairwise comparisons between TRT vs. CON cows on each day indicated that PD was significantly lower in TRT vs. CON cows on day 64 [Cohen's *d* = −1.37, 95% CI (−2.45, −0.29); *p* = 0.02]. Additionally, the contrasts within TRT and CON, indicated that PD decreased significantly in TRT cows [Cohen's *d* = 1.23, 95% CI (0.37, 2.10); *p* = 0.01], and increased significantly in CON cows [Cohen's *d* = 1.23, 95% CI (0.37, 2.10); *p* = 0.01] for the 64-day study. The PD values for TRT vs. CON cows over time can be seen in Figure 3. Observed richness and the Shannon diversity index produced similar results to PD, while Simpson's dominance index indicated no significant differences (Table 2; Supplementary Figures S1– S3).

**Table 2:** Alpha and beta diversities of dairy cows fed a control (CON) diet vs. a treatment (TRT) diet containing a 0.15% dry matter (DM) blend of quebracho tannins.

**Metric**	**Estimated Means**			***p*** **-Values**		
	**TRT** ^a^	**CON** ^b^	**SEM** ^c^	**Treatment**	**Day** ^d^	**Treatment** ^*^Day
**Alpha diversity** ^e^						
Phylogenetic diversity	31.20	30.70	0.88	0.34	0.03	<0.01
Observed richness	452	439	18.10	0.15	0.02	<0.01
Simpson's	0.01	0.01	<0.01	0.73	0.09	0.19
Shannon's	5.32	5.30	0.06	0.49	0.51	0.01
**Beta diversity** ^f^						
Weighted UniFrac	–	–	–	0.16	0.48	0.77

a Tannin treatment (TRT) was top-dressed 0.15% DM blend of quebracho tannins.

b CON (control) cows all received 50 g of dry ground corn as a top dress at each feeding.

c Standard error of the mean is the largest of the two SEMs between the TRT vs. CON groups from the “emmeans” R software output.

d Days 16, 32, and 64, with day 0 as a covariate.

e Alpha diversity metrics calculated with the “alpha” and “pd” functions in R, and analyzed via “lmer.”

f Weighted UniFrac analysis by PERMANOVA.

**Figure 4:**
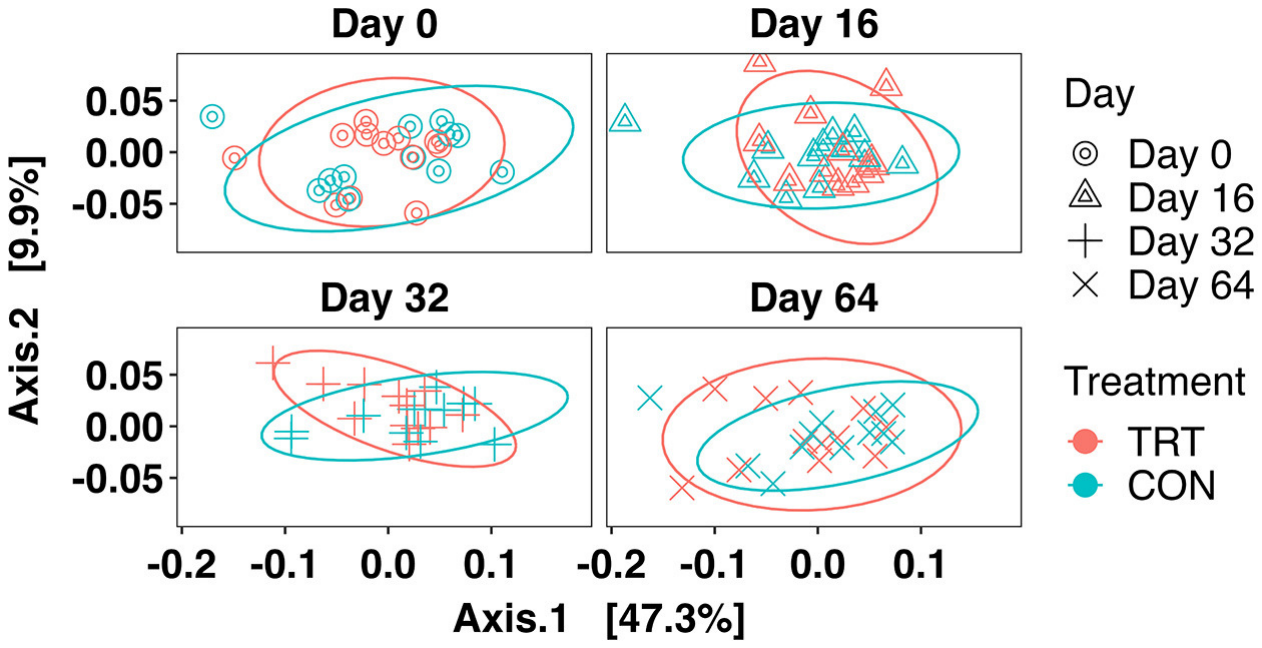
PCoA of the prokaryotic community with weighted UniFrac distances faceted by day for dairy cows fed a control (CON) diet vs. a tannin treatment (TRT) diet containing a 0.15% dry matter (DM) blend of quebracho tannins.

There was no treatment × day interaction effect observed for beta diversity between TRT vs. CON cows when analyzed via PERMANOVA (*r*^2^ = 0.01, *p* = 0.48). The PCoA of Weighted UniFrac distances can be seen in Figure 4, which indicates that neither TRT administration nor day was a major determinant of the fecal prokaryotic community composition. Additionally, the longitudinal effects of TRT vs. CON were assessed with the MicrobiomeStat package, which confirmed that beta diversity did not vary significantly over time (Supplementary Figure S4).

To further investigate the differential relative abundance of individual taxa, the corncob package and the beta-binomial distribution were implemented. The regression model of treatment, controlling for day, returned several species that were differentially abundant and differentially variable (dispersion) between TRT vs. CON cows. These taxa are depicted in Supplementary Figure S5, which includes all taxa with significant initial *p*-values (for day 0) and 95% confidence intervals. Full results of differential abundance and variability are available in Supplementary Table S3.

### 3.3 Fecal chemical properties, greenhouse gas emissions, and metabolites

**Figure 5:**
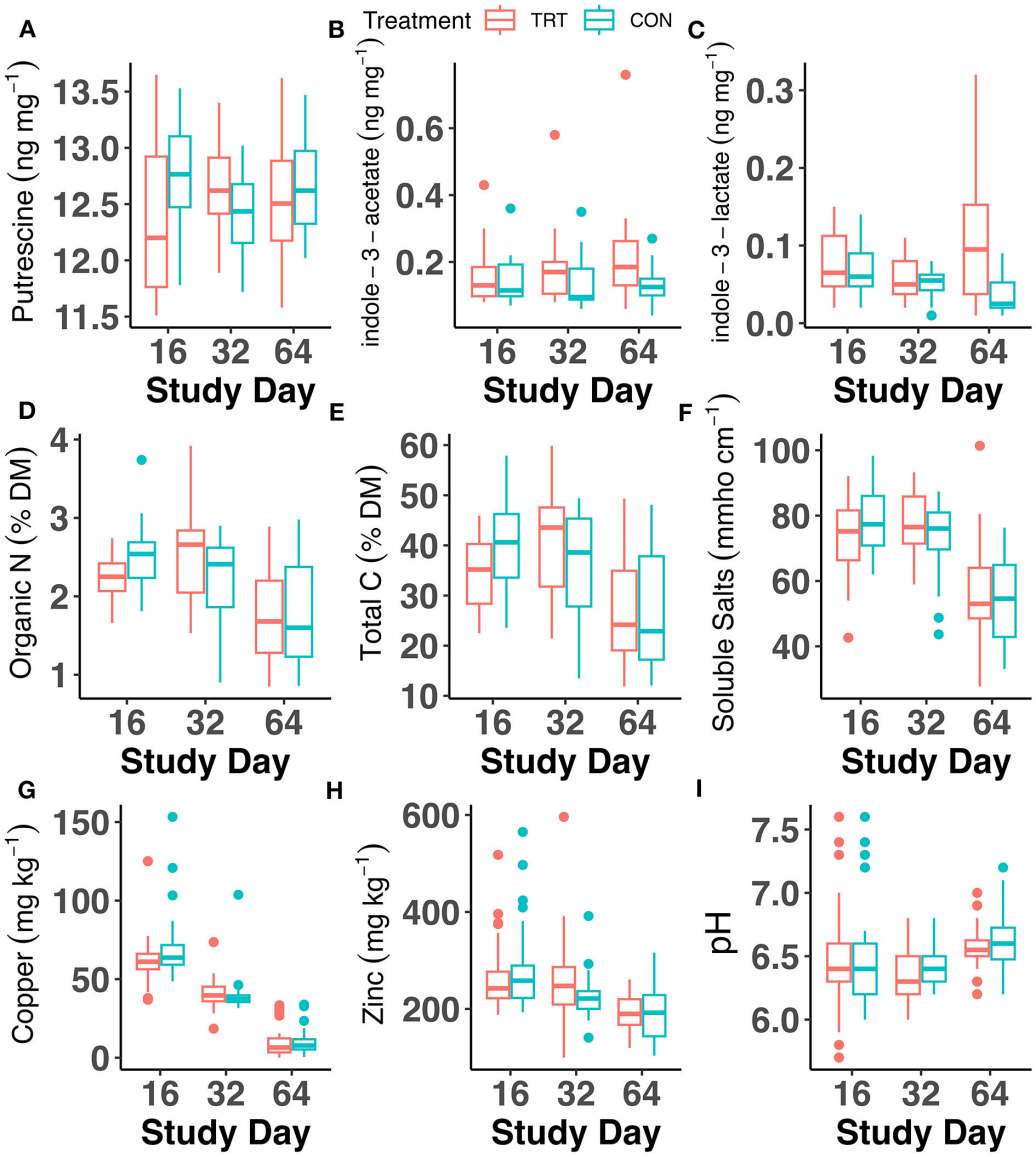
Fecal chemical analysis throughout the 64-day animal study for dairy cows fed a control (CON) diet vs. a tannin treatment (TRT) diet containing a 0.15% dry matter (DM) blend of quebracho tannins. **(A)** Putrescine, **(B)** indole-3-acetate, **(C)** indole-3-lactate, **(D)** organic N, **(E)** total C, **(F)** soluble salts, **(G)** copper, **(H)** zinc, and **(I)** pH.

Analysis of fecal chemical properties indicated significant differences between TRT vs. CON cows, but the largest factor affecting chemical properties was study day (Figure 5; Supplementary Table S4). Significant differences between TRT vs. CON were seen on day 16 for fecal organic N and total C, with both being significantly lower in TRT (*p* < 0.05; Figure 5). However, the significant differences in organic N between TRT vs. CON on day 16 were largely due to a single CON sample value of 3.74% organic N, which, when removed from analysis, led to no significant differences in the contrast. Copper was significantly lower on day 16 in TRT fecal samples vs. CON (*p* < 0.05), and zinc was significantly greater in TRT fecal samples vs. CON on day 32 (*p* < 0.05). No differences were observed in emissions of N_2_O, CH_4_, or CO_2_ for feces samples collected on day 64 and subjected to a 14-day laboratory incubation (*p* > 0.05; Supplementary Figure S6; Supplementary Table S4). The only factor that affected average daily GHGs was day, with fluxes of CO_2_ and CH_4_ peaking around day 3 of the incubation, while fluxes of N_2_O were low until day 14 (Supplementary Table S4; Supplementary Figure S6).

Of the quantified microbial amino acid-derived metabolites, only putrescine, indole-3-acetate, and indole-3-lactate were detected (Figure 5; Supplementary Table S4). Indole-3-lactate exhibited differences between TRT vs. CON fecal samples, with a treatment × day effect (*p* = 0.02) and a significant treatment effect (*p* = 0.01; Figure 5; Supplementary Table S4). Indole-3-lactate values for day 64 were assessed for influential points given the wide range of values observed, and Cook's distance indicated no influential observations at *d* = 0.5. However, an exploratory analysis was initiated with the removal of three indole-3-acetate data points in TRT (0.32, 0.26, and 0.22 ng mg^−1^). The model and statistical analysis were rerun on this reduced dataset, resulting in no significant treatment effect when these values were omitted. No differences between TRT vs. CON were found for putrescine or indole-3-acetate. The differences in indole-3-lactate were evident on day 64, where TRT samples (0.11 ± 0.008 μg kg^−1^) had significantly greater concentrations of indole-3-lactate than CON samples (0.04 ± 0.007 μg kg^−1^; *p* < 0.01).

### 3.4 Correlations among the fecal microbiome and chemical properties

**Figure 6:**
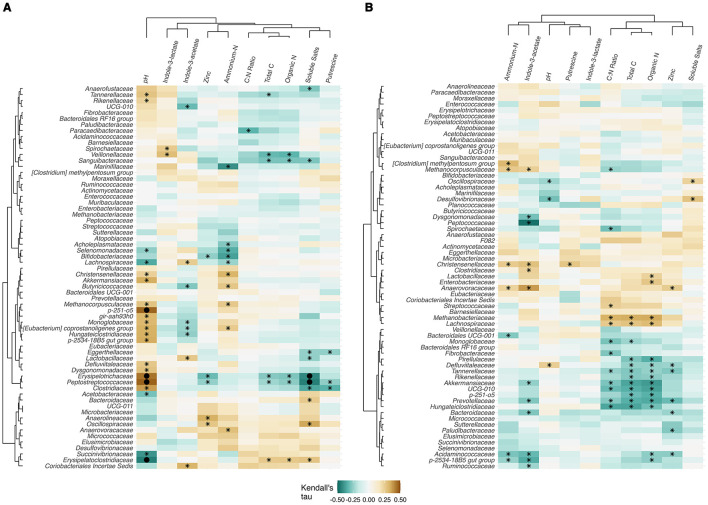
Kendall's tau correlation coefficients of the fecal microbiota of dairy cows and chemical analyses^1^ for **(A)** tannin treatment (TRT), and **(B)** control (CON). The dendrograms were plotted, and *p*- and *q*-values (FDR corrected *p*-values) were calculated with the microViz package. Asterisks indicate *p* < 0.05 and filled circles indicate *q* < 0.05. The dendrogram distances were Euclidean as calculated with the *dist* function, and were clustered with the “Ward.D2” command in the *hclust* function.

Kendall's correlation coefficients were calculated to investigate associations among taxa at the family level, and a subset of the most relevant fecal chemical properties, i.e., those that were found to have significant treatment × day effects (and several that might plausibly be affected by tannin-based feed additives) as determined by *lmer*. As can be seen in Figure 6A, *p-251-o5* (uncultured family of Bacteroidales), *Erysipelotrichaceae, Peptostreptococcaceae*, and *Erysipelatoclostridiaceae* exhibited correlations in TRT samples with *q* < 0.05 for pH, and *q* < 0.05 for soluble salts (*Erysipelotrichaceae* and *Peptostreptococcaceae*). No *q*-values (FDR corrected *p*-values) were significant among CON samples (Figure 6B). There were numerous correlations among CON sample microbial communities and total C and organic N; however, none were significant at *q* < 0.05. The correlations in CON samples demonstrated a pattern of total C, organic N, and C:N ratio correlating with family (*p* < 0.05), while the relationships among family and chemical properties were less prevalent in the TRT heatmap, with the exception of several associations previously mentioned.

## 4 Discussion

In this study of a tannin-based feed additive in a dairy cattle diet, we saw various effects on the microbial community and fecal chemical composition that could indicate a functional shift in the microbiome. However, since our results were marked by only modest effect sizes over time, the results need to be interpreted accordingly. With feed intake being similar between TRT vs. CON cows over the course of the study (Supplementary Table S5), the differences cannot be attributed to a drop in DM consumption in treatment animals, which can occur at high CT doses (Aboagye and Beauchemin, 2019).

The PD differences over the 64-day study (Figure 3) require a cautious interpretation given the significant treatment × day interaction. There was a significant decrease in TRT PD between days 16 and 64, while CON PD significantly increased. Our results for PD bear some similarity to those of Fagundes et al. (2021) who documented a decrease in total bacterial abundance over 60 days when feces from cows fed condensed tannins at 1.25% DM were soil amended. Unlike in our study, the authors also documented an increase in fecal N excretion; however, they were fed tannins at a substantially higher dose than in our study. The native microbial diversity in the ruminant GIT is well-known to vary significantly in the transition from early-life to maturity (Meale et al., 2017), in response to changes in diet (Gruninger et al., 2019) and in changes in physiologic stress (Li et al., 2020). Therefore, identifying meaningful biologically relevant effects of a feed additive over time can be quite difficult when numerous environmental variables are at play, treatments are administered at low doses and/or treatment effects are presumably at the detection limit. Still, the completely randomized block design in our study should have accounted for these extraneous variables. Then, given no significant differences in animal performance metrics (Supplementary Table S5) or fecal CH_4_ production over the course of the study (Supplementary Figure S6), the response here suggests that the shift in diversity had no functional effect on lower GIT parameters associated with N partitioning and/or metabolism. Our results are similar to Díaz Carrasco et al. (2017), who also found a significant decrease in species richness when cattle were fed a quebracho-based feed additive; however, unlike our study, they also found a significant increase in pH in the tannin-fed rumen fluid samples.

Our study found that fecal organic N was lower for TRT vs. CON cows on day 16, but that this effect was due in part to an influential point in CON cows. Removing that point led to no significant differences. The majority of studies on the topic find that tannin inclusion in the diet leads to an increase in fecal N, likely through the mechanism of dietary tannins inhibiting N bioavailability to rumen microbes (Aguerre et al., 2016; FAO, 2023). Because our results are not entirely in accordance, a cautious explanation is warranted. It is possible that at low concentrations, tannins are more susceptible to pH (Hagerman et al., 1992) or polarity modification (Hagerman and Klucher, 1986), which can reduce the strength of tannin-protein bonds (Hagerman et al., 1992). It is also possible that at low doses, TRT does not bind to enough N to lead to a detectable shift of N from urine to feces. To the best of our knowledge, Carvalho et al. (2024) is the most recent publication on a similar quebracho feed additive being administered to cattle (also at 0.15% DM). The authors found no differences in animal performance between control and feed additive groups, which could be seen as corroborating the results herein, that CT at 0.15% DM did not substantially shift enteric parameters.

We also found an increase in the concentration of indole-3-lactate in TRT vs. CON cow samples, a finding that warrants further investigation. Three TRT samples with substantially greater concentrations of indole-3-lactate contributed to the statistical significance of our results; however, these samples passed the influential point test (Cook's distance < 0.5) and were therefore not considered true outliers. This finding demonstrates the potential for highly individualized interactions between taxa and indole-3-lactate or metabolite production and also highlights that extreme, but rare responses may not be detected when sample sizes are low. The two families we found correlated with indole-3-lactate, *Spirochaetaceae* and *Veillonellaceae*, are not typically reported as key producers of the metabolite. While *Spirochaetaceae* and *Veillonellaceae* may not directly produce indole-3-lactate, they could influence its levels indirectly through interactions within the gut microbial community. For example, *Veillonella* spp. are known for fermenting lactate into short-chain fatty acids such as propionate, playing a role in the GIT metabolic network (Zhang and Huang, 2023). Current literature, to the best of our knowledge, provides no direct evidence linking *Spirochaetaceae* to the production of indole-3-lactate; however, various genera in the Spirochetes phylum contribute significantly to fiber digestion and can ferment plant polymers such as pectin, xylan, and arabinogalactan (Paster and Canale-Parola, 1982). These bacteria could play a role in modulating the gut environment, influencing factors such as pH and substrate availability, which could indirectly affect the production of various metabolites such as indole-3-lactate.

The importance of amino acid metabolism on health is well-reported in dairy cattle (Yang et al., 2022); however, the trace metabolite indole-3-lactate, known for its role in bacterial quorum sensing, i.e., inter- and intra-kingdom population monitoring (Shatova and Shestopalov, 2023) and in stimulating host antioxidant status (Ehrlich et al., 2020), has not been extensively studied in dairy cattle (Yang et al., 2022). Numerous bacterial species produce indole-3-lactate in the GIT, including *Clostridium* spp., *Peptostreptococcus* spp., *Lactobacillus* spp., *Bifidobacterium* spp., and *Bacteroides* spp. (Gasaly and Gotteland, 2022). It has been demonstrated that indole-3-lactate produced in the GIT is absorbed by the intestinal epithelium where it can activate a wide array of host anti-inflammatory pathways, including the attenuation of LPS-induced NF-κB activity, attenuation of TNF-α- and LPS-induced increases in pro-inflammatory cytokines, and increased expression of aryl hydrogen receptor target genes (Ehrlich et al., 2020; Gasaly et al., 2021; Shatova and Shestopalov, 2023). Gasaly and Gotteland (2022) indicated that tannins affect the bacterial degradation of individual amino acids, which can lead to increased production of various indoles. Various members of the microbial community respond to CT presence by degrading amino acids in ways that produce unique metabolites, presumably due to the new chemical structures formed among tannins and bound amino acids. The mechanisms by which this occurs are not well-defined currently, but future research should investigate this topic and the extent to which tannins confer anti-inflammatory benefits in dairy cattle.

The trends in copper and zinc among TRT and CON samples also require further investigation and highlight the dynamic and complex interactions of the gut microbial community and essential trace elements. Copper is an essential cofactor for numerous mammalian enzymes involved in antioxidant response, maintenance of membranes and DNA, and ATP production (Nargund et al., 2015; Dalecki et al., 2017) and is also used by bacteria in various metabolic pathways (Andrei et al., 2020). Copper availability also modulates the microbial community through direct antimicrobial activity (Pajarillo et al., 2021). Zinc is also an essential cofactor, required for over 300 enzymes and 1,000 transcription factors (McCall et al., 2000). Zinc has a well-demonstrated ability to confer host resistance to bacterial infection and can also directly impact bacteria by disrupting bacterial cellular processes upon absorption (Pajarillo et al., 2021). The ability of tannins to chelate these metals can limit their availability for absorption by the ruminant host or promote bioavailability depending on the nature of the chelating agent (Gowda et al., 2024). Chelation and reduced bioavailability can also confer benefits by reducing metal reactivity. For example, excess free copper in the gut acts as a prooxidant, leading to increased inflammatory markers (Zhang et al., 2017). That both copper and zinc decreased in TRT and CON cows over the 64-day study (Figure 5) leads to the conclusion that an interaction between diet and animal absorption was occurring. The copper content of the diet averaged 16.3 ± 2.8 ppm while the zinc content averaged 72.3 ± 3.8 ppm (with the greatest value for zinc occurring on day 64; Supplementary Table S6). As such, the decreases in fecal copper and zinc are quite surprising. Copper and zinc both undergo complex absorption dynamics in the ruminant GIT, depending on the concentrations of other elements (e.g., S, Fe, Mo, and Mn) and are not absorbed simply in proportion to intake (Suttle, 2016). In ruminants, understanding of these regulatory mechanisms is incomplete; however, marked differences in absorption dynamics over time and in relation to lactation stage have been documented (Daniel et al., 2023). Future research on CT chelation of metals in the GIT could investigate the interaction of CT with the inherent lactation-stage absorption dynamics.

We also observed several associations among prokaryotic families and fecal chemical properties that differed between TRT vs. CON cows (Figure 6). Several families in TRT correlated positively with pH, and two correlated negatively with soluble salts. These species could be investigated further for their roles in stabilizing pH in the GIT. One family significantly associated with pH in our study, *Erysipelotrichaceae*, was found by Myer et al. (2015) to be differentially abundant in fecal samples among cattle with different feed intakes and average daily gain. The *Peptostreptococcaceae* family was also found in our study to correlate with pH; this family is documented as containing high ammonia producers (Congiu et al., 2024) and is associated with low nitrogen use efficiency of the animal (Alves et al., 2021). The results here indicate that several species could be investigated more specifically for their susceptibility to tannins and/or for improving N efficiency of the animal by promoting efficient N use in the microbial community. We hypothesize that these associations indicate a mechanistic link between CT, ammonia production, and pH. Given the buffering capacity of the rumen environment, a substantial increase in ammonia production could occur without varying the pH significantly. This could have downstream effects on manure fertilizer N availability. Ingold et al. (2021) found that tannin feed additives affect manure mineralization dynamics only when amended to an alkaline soil, and it is generally thought that tannins bind to organic N at neutral to alkaline pH (Aboagye and Beauchemin, 2019). Therefore, the families we found to correlate with TRT and with pH might be worth investigating as potentially beneficial soil microbes aiding in manure N availability in soil. While TRT did not shift individual taxa to a great extent, we see a potential for this product as a GIT modifier, with the potential to reduce fecal N excretion and potentially improve the anti-inflammatory status of the animal, topics that should be investigated in future studies.

The utility of tannin-based feed additives in modifying the microbial community has been demonstrated in previous studies, albeit with considerable variability. For example, Díaz Carrasco et al. (2017) fed quebracho and chestnut tannins at 0.2% DM to dairy cattle for two 12-day periods, and found a significant increase in the Firmicutes/Bacteroidetes ratio of rumen fluid samples for treatment-fed (similar ingredients to our TRT) cattle. The authors also found a decrease in Shannon's diversity index in TRT relative to CON in that study. Our study did not find a significant difference in the Firmicutes/Bacteroidetes ratio (*p* = 0.76; Table 1); however, comparisons between the present study and that of Díaz Carrasco et al. (2017) could be seen as evidence for a variable effect of TRT through the GIT, due to differences between the rumen and lower GIT (e.g., pH in the rumen vs. intestines). Since Díaz Carrasco et al. (2017) did not measure fecal microbial communities, that possibility cannot be confirmed, but could be investigated in the future. Notably, enteric responses to tannin-based feed additives are known to be highly variable at tannin concentrations < 2% DM (Jayanegara et al., 2012). As such, efficacy may depend on numerous lurking variables. Lower doses, like those in our study, make administering the feed additive less costly and minimize the risk of reduced feed intake and reduced digestibility observed when tannins are fed at high doses (Aboagye and Beauchemin, 2019), but potentially do not deliver consistent binding of organic N.

Beyond effects on N partitioning, CTs are being considered for their potential to reduce enteric and, more recently, manure, GHG emissions. For example, Fagundes et al. (2020) fed CT to Zebu cattle at 1.25 and 2.5% DM and investigated effects on manure CH_4_. The authors found no significant decrease in manure emissions relative to control manure from cattle fed CT at 0% DM. These results are similar to our lab incubation, where our TRT feed additive at 0.15% DM did not affect fecal CH_4_, N_2_O, or CO_2_ (Supplementary Table S4; Supplementary Figure S6). However, similar to Fagundes et al. (2021), we saw a decrease in bacterial diversity in fecal samples throughout the feeding trial. This decrease could potentially act as an environmental remediator, as indicated by Fagundes et al. (2021), their treatment slowed the release of soil C and N when CT-fed cattle manure was soil amended. The impacts of tannins on environmental parameters are historically well-studied from a soil science perspective (Kraus et al., 2003); less understood are the dynamic and complex effects of changes in cattle diets on manure characteristics and C and N cycling. As climate-smart agriculture becomes increasingly adopted globally, demand for manure fertilizers will likely increase as these products are seen as less energy-intensive than conventional fertilizers and their use is considered a waste repurposing, i.e., well-managed manure fertilizers are assets rather than environmental liabilities. However, the still unanswered questions about manure C and N cycling need to be addressed.

## 5 Conclusion

The present study of a 0.15% DM blend of quebracho tannins entails an important extension to the current understanding of CT feed additive effects on the fecal microbial community and associated fecal chemical properties. It is likely that the adoption of feed additives will increase in the near future, along with interest in the mitigation of ruminant GHG emissions. However, the successes of CT treatments *in vitro* do not always align with the results of *in vivo* trials, owing to factors such as CT source, dose, and animal life stage. If the effects of tannins are minimal in the lower GIT, or do not substantially improve animal health or production, then these products may be best suited for addressing short-term complications such as dysbiosis. Increased sampling frequency and testing under various diet conditions would be useful in longer-term studies to confirm this. While several studies have demonstrated that CT-based feed additives confer benefits to GIT metabolism, the results of the present study should be confirmed with an approach targeting tannin impacts on microbial amino acid metabolism. The severity of the climate crisis and the need for easily implementable solutions warrant that investigation into CT-based products should continue with the goal of discovering effective and replicable solutions.

## Data Availability

The raw DNA sequences are available in the NCBI Sequence Read Archive (SRA), under accession number PRJNA1106901.

## Footnotes

^1^Asterisk indicates *p* < 0.05, not FDR adjusted. Filled circle indicates FDR adjusted *p* < 0.05.
