# Supplementary material for: Limited effects of tannin supplementation on the dairy cattle fecal microbiome with modulation of metabolites
Source: Front Microbiol. 2025 Jun 10;16:1570127. doi: 10.3389/fmicb.2025.1570127 (PMC12186457; doi:10.3389/fmicb.2025.1570127)

Supplementary Material

Limited Effects of Tannin Supplementation on the Dairy Cattle Fecal Microbiome with Modulation of Metabolites

Matthew L Klein^1^, Christian B Erickson^2^, Conor J McCabe^1^, Laibin Huang^3^, Jorge L Mazza Rodrigues^2,4^, Frank M Mitloehner^1*^

^1^ Department of Animal Science, University of California, Davis, Davis CA, 95616, USA

^2^ Department of Land, Air, & Water Resources, University of California, Davis, Davis CA, 95616, USA

^3^ Department of Biology, Saint Louis University, St. Louis, Missouri, 63103, USA

^4^ Environmental Genomics and Systems Biology Division, Lawrence Berkeley National Laboratory, Berkeley, CA, 94720, USA

*** Correspondence:**Frank Mitloehner
fmmitloehner@ucdavis.edu

**Supplementary Table S3.** Differential abundance results utilizing the beta-binomial distribution

|  |  | | | | | | | | | | | | | | |  |
| --- | --- | --- | --- | --- | --- | --- | --- | --- | --- | --- | --- | --- | --- | --- | --- | --- |
|  | | Abundance^2^ | | | | |  | | | | Dispersion | | | | | |
| **Phyla** / *Genus or Species*^1^ | | Day 16 | Day 32 | | Day 64^3^ | | | SEM^4^ |  | | | Day 16 | Day 32 | Day 64 | SEM | |
| **Firmicutes** | |  | |  |  |  | | | |  |  |  |  |  |  |  |
| *Lachnospiraceae NK4A136 group* | | 0.06 | 0.84 | | 0.93 | | | 0.34 |  | | | <0.001* | 0.05* | 0.21 | 1.01 | |
| *Lachnospiraceae NK3A20 group* | | 0.59 | 0.33 | | 0.53 | | | 0.24 |  | | | 0.13 | 0.33 | 0.01* | 0.84 | |
| *Limosilactobacillus* | | 0.15 | 0.20 | | 0.30 | | | 0.65 |  | | | 0.06 | 0.01* | <0.001* | 1.44 | |
| *Family XIII UCG-001* | | 0.46 | 0.13 | | 0.36 | | | 0.58 |  | | | 0.07 | 0.26 | 0.05 | 1.63 | |
| *Romboutsia* | | 0.27 | 0.23 | | 0.71 | | | 0.25 |  | | | 0.20 | 0.44 | 0.83 | 0.84 | |
| *Lachnospiraceae UCG-009* | | 0.67 | 0.67 | | 0.01* | | | 0.18 |  | | | 0.65 | 0.21 | 0.01* | 0.96 | |
| *Erysipelatoclostridium* | | 0.39 | 0.68 | | 0.45 | | | 0.31 |  | | | 0.79 | 0.61 | 0.49 | 1.34 | |
| *Peptococcaceae* | | 0.73 | 0.30 | | 0.88 | | | 0.69 |  | | | 0.22 | 0.49 | 0.85 | 1.09 | |
| *Cellulosilyticum* | | 0.79 | 0.09 | | 0.60 | | | 0.49 |  | | | 0.45 | 0.46 | 0.08 | 0.94 | |
| *Incertae Sedis* | | 0.83 | 0.35 | | 0.46 | | | 0.71 |  | | | 0.53 | 0.12 | 0.18 | 1.12 | |
| **Actinobacteriota** | |  |  | |  | | |  |  | | |  |  |  |  | |
| *Bifidobacterium* | | 0.32 | 0.59 | | 0.76 | | | 2.28 |  | | | 0.36 | 0.45 | 0.76 | 2.54 | |
| **Proteobacteria** | |  |  | |  | | |  |  | | |  |  |  |  | |
| *Succinivibrio* | | 0.46 | 0.82 | | 0.53 | | | 0.86 |  | | | 0.17 | 0.61 | 0.004* | 1.20 | |

^1^ Taxa included all had at least one significant *p*-value in the full model results i.e., intercepts, single factor levels, included here are daily pairwise comparisons between TRT and CON.

^2^ TRT treatment was included in the diet as a top dress at 0.15% of dietary dry matter. Tannin composition included: blend of tannins. Control (CON) cows all received 50 g of dry ground corn as a top dress at each feeding.

^3^ Day included the three timepoints fecal measurements were recorded post treatment administration i.e., 16-, 32-, and 64-days.

^4^ Standard error of the mean (SEM) is the largest of the SEMs between the TRT and CON groups from the *corncob* model output.

* Denotes FDR corrected p < 0.05.

**Supplementary Table S4.** Fecal chemical properties of dairy cows fed a control (CON) diet vs. a tannin treatment (TRT) diet containing a 0.15% dry matter (DM) blend of quebracho tannins.

|  | **Estimated Means** | | | **P values** | | |
| --- | --- | --- | --- | --- | --- | --- |
| **Metric** | **TRT^1^** | **CON^2^** | **SEM^3^** | **Treatment** | **Day^4^** | **Treatment × Day** |
| Putrescine, ng/mg | 12.54 | 12.61 | 0.161 | 0.87 | 0.13 | 0.29 |
| Indole-3-acetate, ng/mg | 0.19 | 0.14 | 0.235 | 0.10 | 0.75 | 0.49 |
| Indole-3-lactate, ng/mg | 0.08 | 0.05 | 0.02 | 0.01 | 0.25 | 0.02 |
| Organic N, % DM | 2.16 | 2.15 | 0.14 | 0.77 | <0.01 | <0.01 |
| Total C, % DM | 34.05 | 34.19 | 2.51 | 0.75 | <0.01 | <0.01 |
| C:N | 14.78 | 14.89 | 0.37 | 0.33 | <0.01 | 0.09 |
| Phosphorus, % DM | 2.04 | 2.00 | 0.12 | 0.65 | <0.01 | 0.77 |
| Potassium, % DM | 0.86 | 0.87 | 0.05 | 0.69 | <0.01 | 0.19 |
| Iron, ppm | 1272.00 | 1165.33 | 156.00 | 0.41 | <0.01 | 0.75 |
| Zinc, ppm | 239.33 | 232.00 | 16.50 | 0.51 | <0.01 | 0.051 |
| Copper, ppm | 37.52 | 39.88 | 3.00 | 0.19 | <0.01 | 0.062 |
| Soluble Salts, mmho/cm | 68.63 | 69.33 | 3.17 | 0.77 | <0.01 | 0.02 |
| pH | 6.44 | 6.51 | 0.08 | 0.31 | <0.01 | 0.83 |
| Moisture, % | 83.77 | 83.50 | 0.89 | 0.71 | <0.01 | 0.06 |
| Average daily N_2_O, µg kg^-1^ | 1.27 | -5.24 | 8.34 | 0.55 | 0.55 | 0.96 |
| Average daily CH_4_, µg kg^-1^ | 5.95 | 5.56 | 0.97 | 0.61 | 0.01 | 0.90 |
| Average daily CO_2_, mg kg^-1^ | 313 | 313 | 12.7 | 0.60 | <0.01 | 0.71 |

^1^Tannin treatment (TRT) was a top-dressed 0.15 % DM blend of quebracho tannins.

^2^CON (Control) cows all received 50 g of dry ground corn as a top dress at each feeding.

^3^Standard error of the mean is the largest of the two SEMs between the TRT and CON groups

from the *emmeans* R software output.

^4^Day included the three timepoints, day 16-, 32-, and 64, with day 0 as a covariate.

**Supplementary Table S5**

Dairy cattle feed intake and milk yield averaged over the course of the study between cows fed a control (CON) diet vs. a tannin treatment (TRT) diet containing a 0.15% DM blend of quebracho tannins

| Variable | Estimated Mean | | | P-Value | | |  |
| --- | --- | --- | --- | --- | --- | --- | --- |
|  | CON^1^ | TRT^2^ | SEM^3^ | Treatment | Period^4^ | Treatment x Period | |
| Milk Yield (kg/d) | 38.9 | 37.3 | 1.1 | 0.31 | <0.01 | 0.47 | |
| Intake (kg/d) | 28.4 | 28.5 | 0.38 | 0.88 | <0.01 | 0.24 | |

^1^CON (Control) cows all received 50 g of dry ground corn as a top dress at each feeding.

^2^Tannin treatment (TRT) was included in the diet as a top dress at 0.15% of dietary dry matter. Tannin composition included: blend of tannins

^3^Standard error of the mean is the largest of the two SEMs between the TRT and CON groups

^4^Period included four timepoints where head-chamber measurements were recorded and included 16-, 32-, 48-, and 64-days post treatment initiation.

## Supplementary Figures

## Figure S1

##
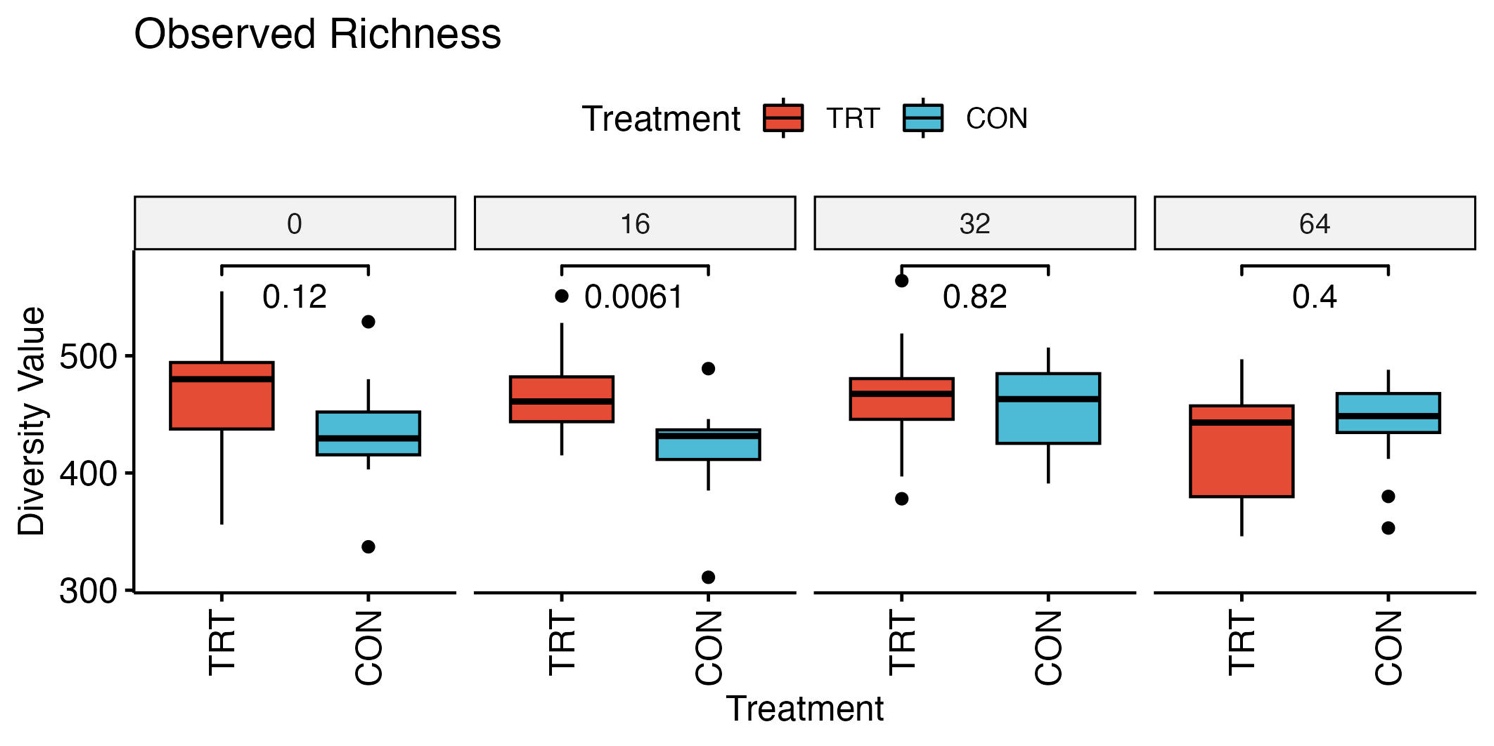


## Figure S2


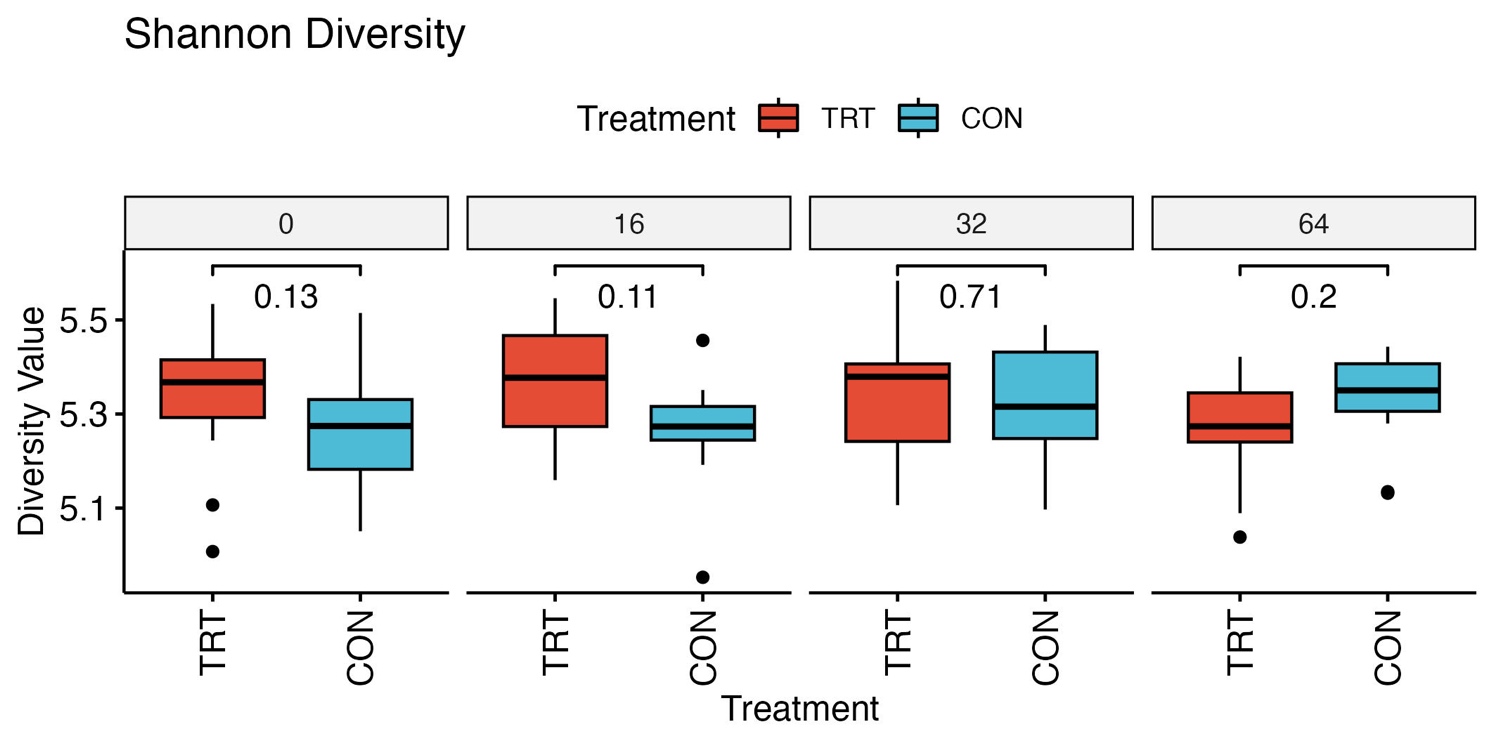


**Figure S3**

##
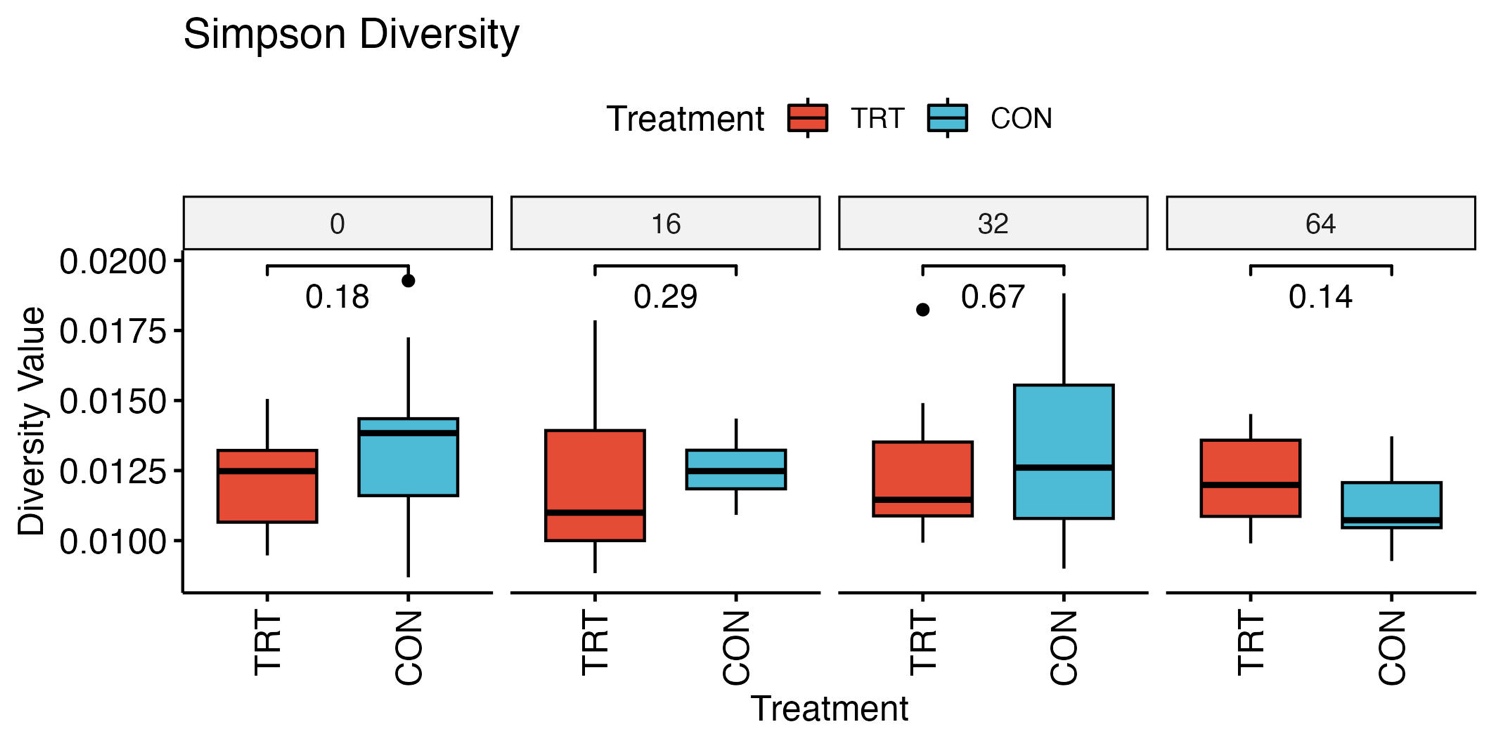


**Figure S4.** *P* values and coefficients for beta-diversity results from the mixed model analysis performed with the MicrobiomStat package in R of the prokaryotic community in fecal samples from dairy cows fed a control (CON) diet vs. a tannin treatment (TRT) diet containing a 0.15% dry matter (DM) blend of quebracho tannins at 16-, 32-, and 64-days post TRT administration.

**
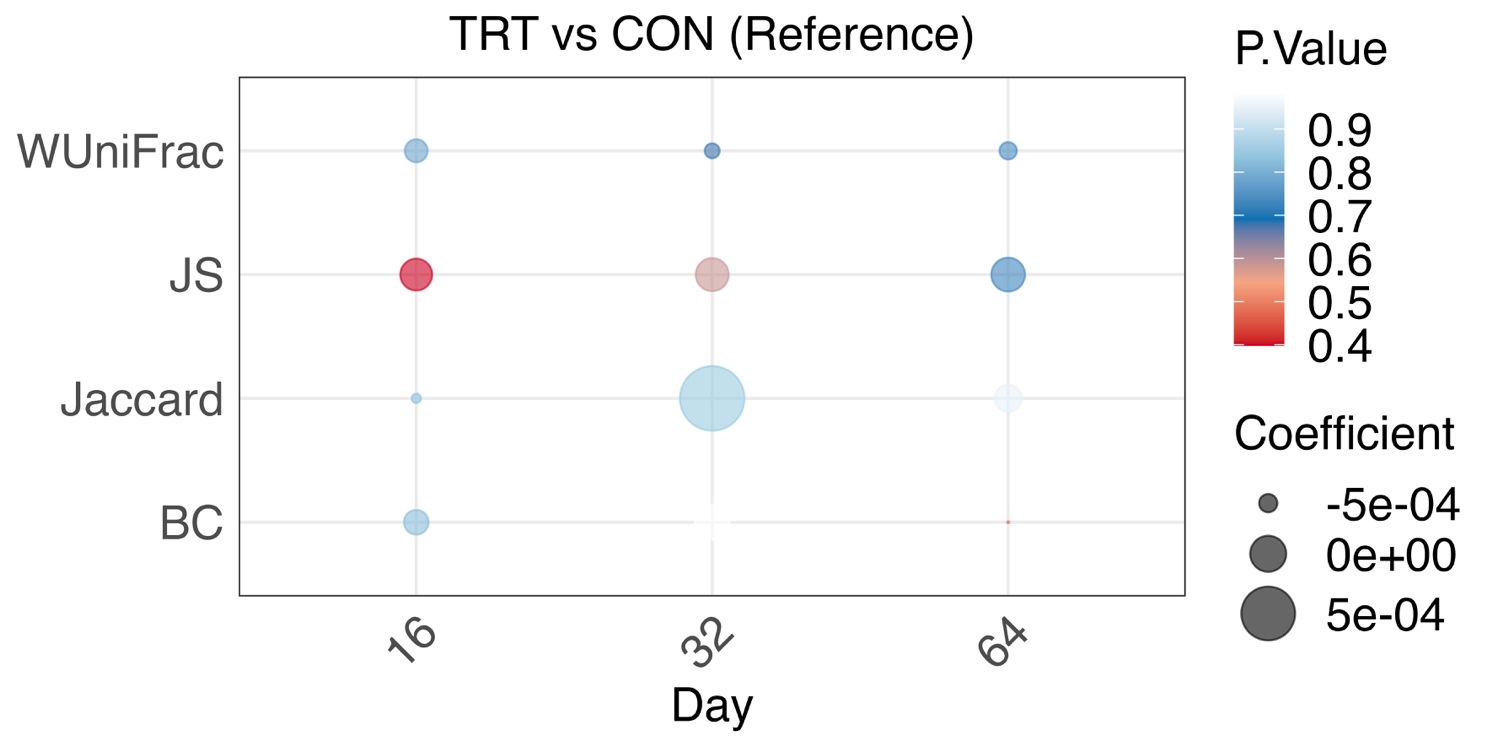
**

**Figure S5.** Differential relative abundance of the prokaryotic community in fecal samples from dairy cows fed a control (CON) diet vs. a tannin treatment (TRT) diet containing a 0.15% dry matter (DM) blend of quebracho tannins at 16-, 32-, and 64-days post TRT administration^1^.

**
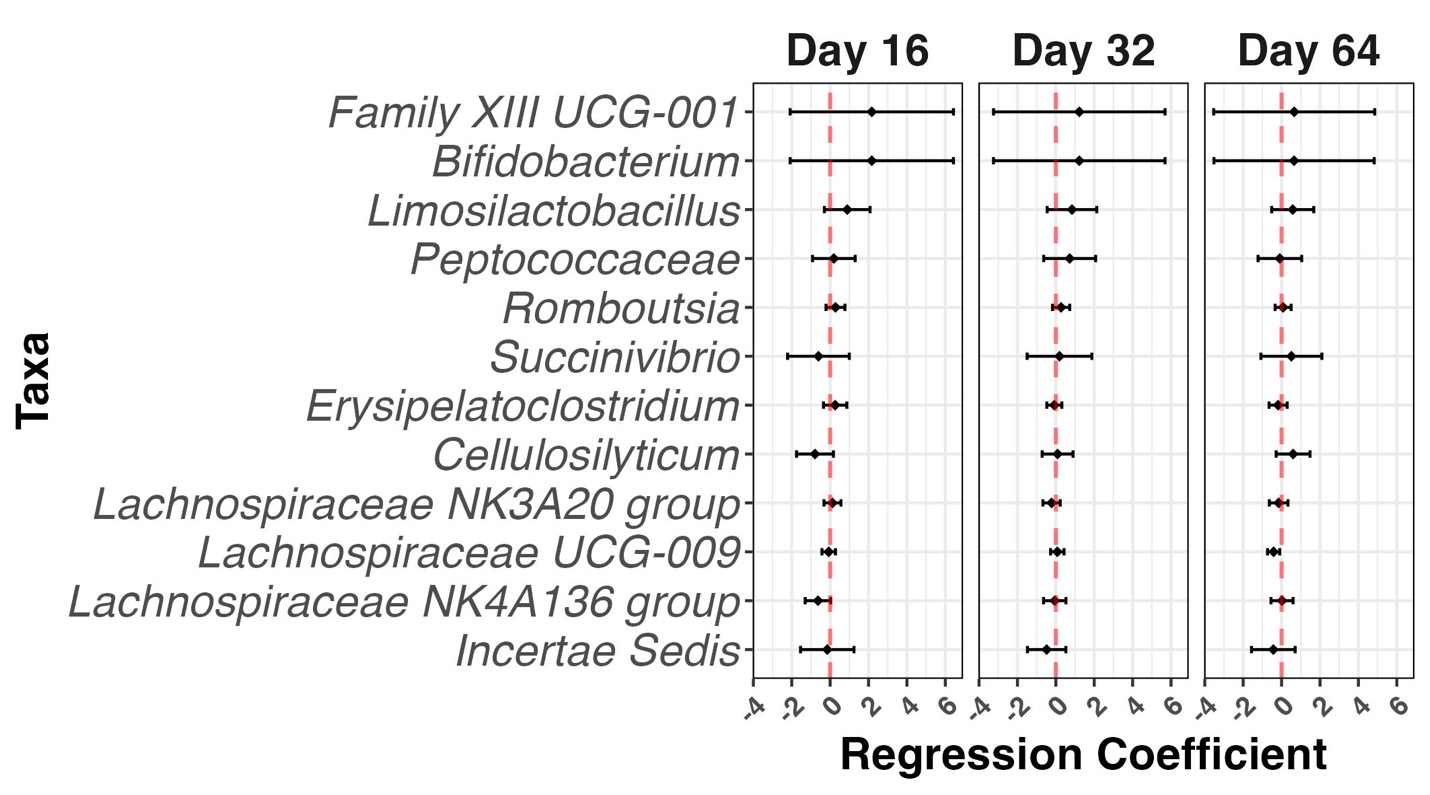
**

^1^Bars represent 95% confidence intervals generated through a beta-binomial regression model. Positive regression coefficient values indicate increased abundance in TRT relative to CON. Taxa depicted had significant intercept *p* values, indicating differences on day 0.

**Figure S6** Dynamic daily fluxes of (A) CO_2_, (B) N_2_O, and (C) CH_4_ over the course of a 14-day laboratory incubation with feces collected from dairy cattle fed either a control (CON) diet or a tannin treatment (TRT) diet containing a 0.15% dry matter (DM) blend of quebracho tannins.


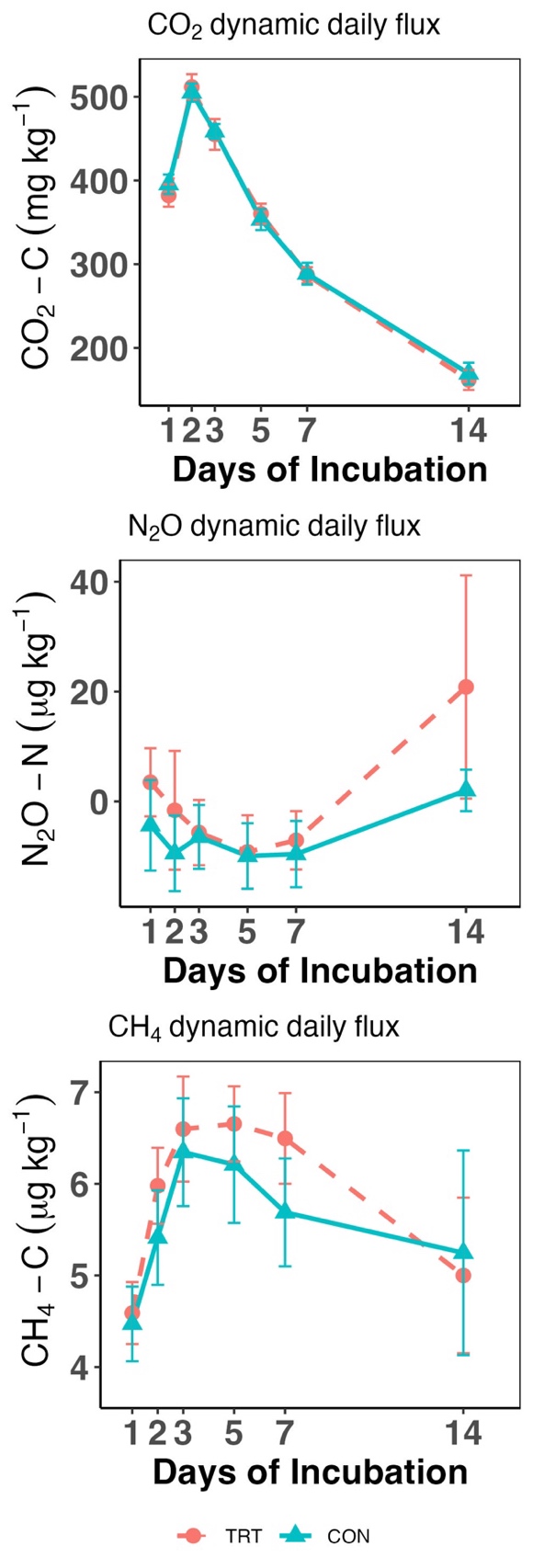


**Supplementary Table S6.** Diet nutrient analysis for the baseline diet, midpoint, and end-of-study diet fed to all TRT and CON cows.


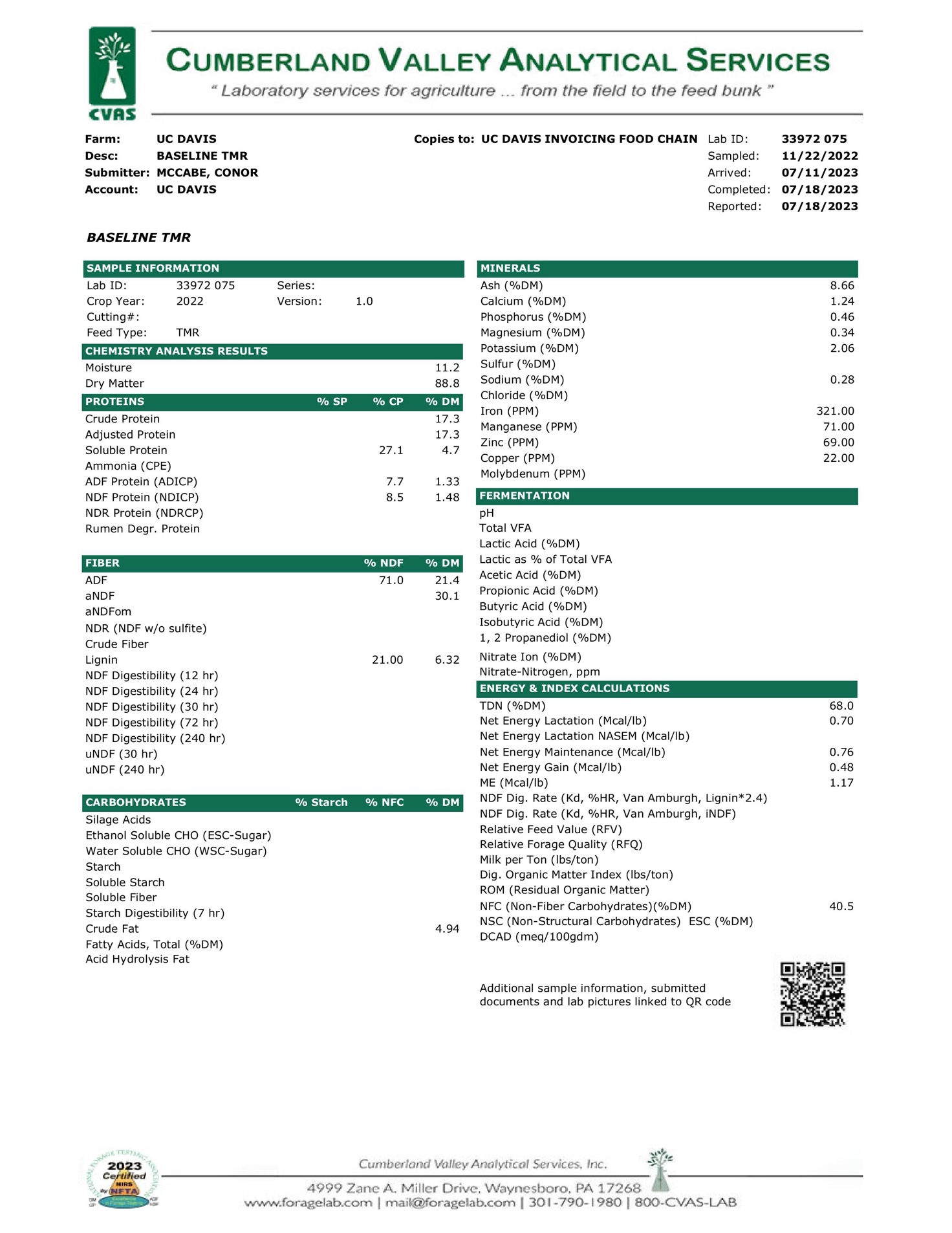


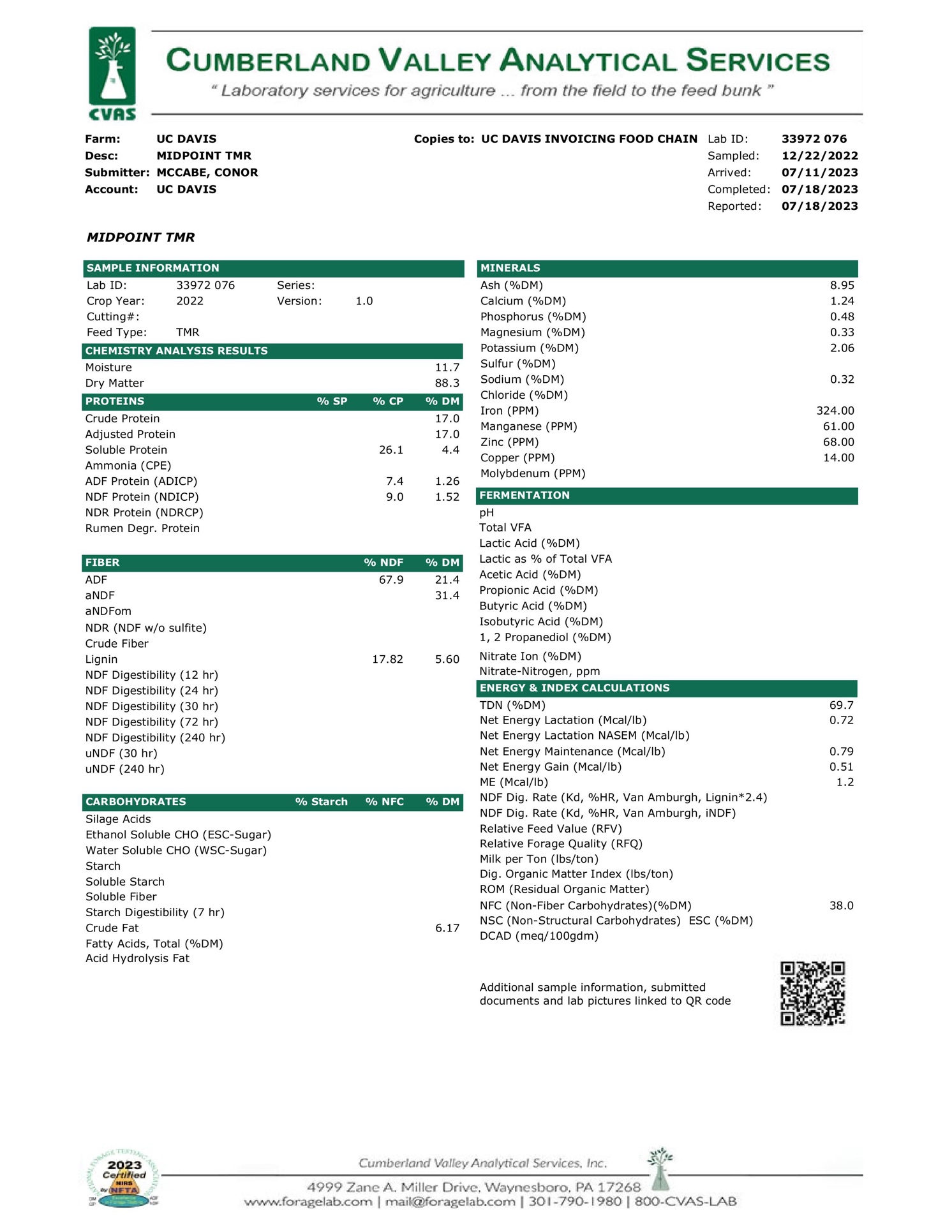


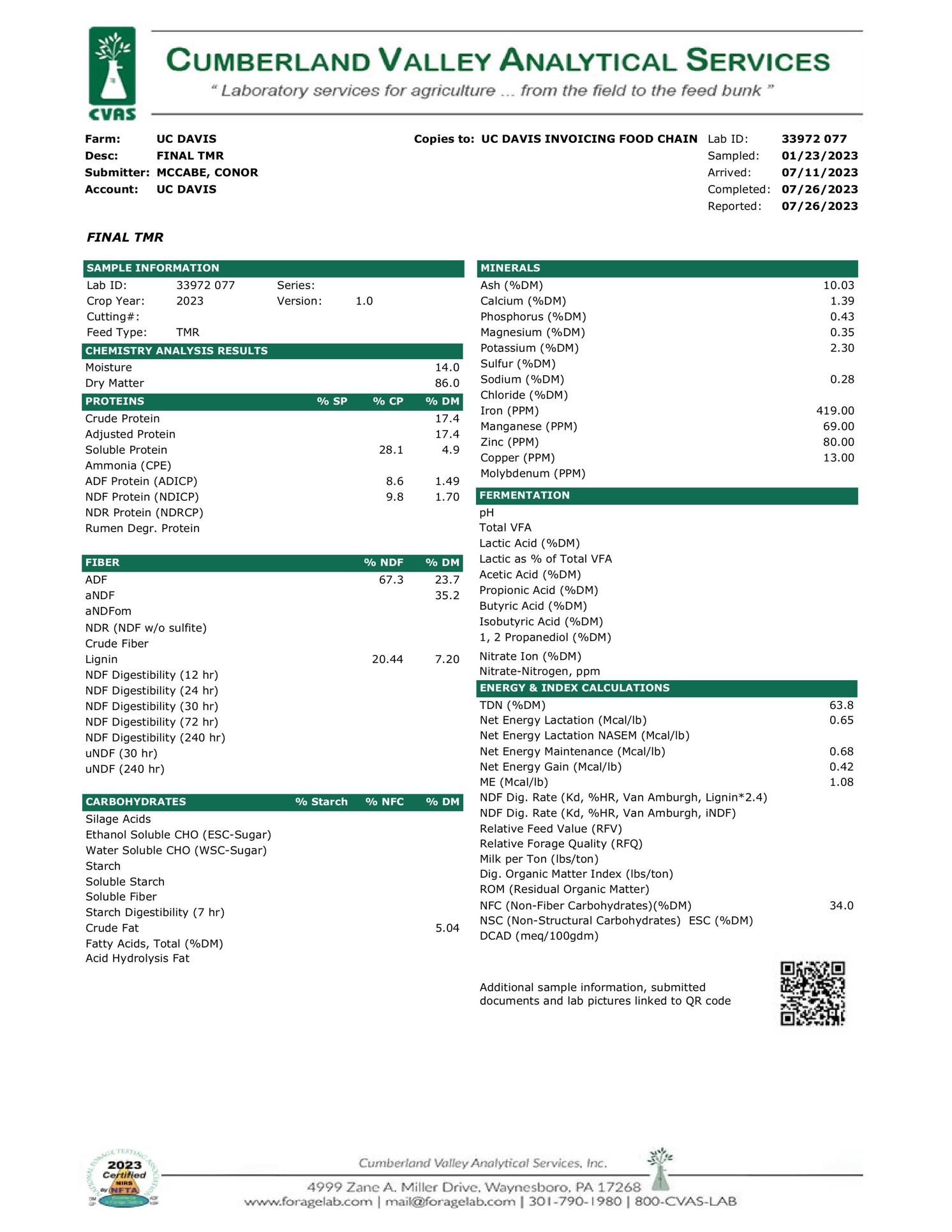

Supplement: Supplementary file 3 [file Supplementary_file_3.docx]
